# Prognostic Value of Mitral Regurgitation in Patients with Primary Hypertrophic Cardiomyopathy

**DOI:** 10.3390/medicina59101798

**Published:** 2023-10-09

**Authors:** Milorad Tesic, Lazar Travica, Vojislav Giga, Ivana Jovanovic, Danijela Trifunovic Zamaklar, Dejana Popovic, Djordje Mladenovic, Marija Radomirovic, Jelena Vratonjic, Nikola Boskovic, Srdjan Dedic, Olga Nedeljkovic Arsenovic, Srdjan Aleksandric, Stefan Juricic, Branko Beleslin, Ana Djordjevic Dikic

**Affiliations:** 1 Clinic for Cardiology, University Clinical Center of Serbia, 11000 Belgrade, Serbia; lazartravica18@gmail.com (L.T.); voja2011@yahoo.com (V.G.); danijelatrif@gmail.com (D.T.Z.); marija3793@gmail.com (M.R.); stefan.juricic@gmail.com (S.J.); skali.ana7@gmail.com (A.D.D.); 2 Faculty of Medicine, University of Belgrade, 11000 Belgrade, Serbia; olganedeljkovic@gmail.com; 3 Faculty of Pharmacy, University of Belgrade, 11000 Belgrade, Serbia; 4 Center for Radiology and Magnetic Resonance Imaging, University Clinical Center of Serbia, 11000 Belgrade, Serbia

**Keywords:** prognosis, mitral regurgitation, hypertrophic cardiomyopathy, echocardiography

## Abstract

*Background and Objectives*: Mitral valve pathology and mitral regurgitation (MR) are very common in patients with hypertrophic cardiomyopathy (HCM), and the evaluation of mitral valve anatomy and degree of MR is important in patients with HCM. The aim of our study was to examine the potential influence of moderate or moderately severe MR on the prognosis, clinical presentation, and structural characteristics of HCM patients. *Materials and Methods*: A prospective study examined 176 patients diagnosed with primary asymmetric HCM. According to the severity of the MR, the patients were divided into two groups: Group 1 (*n* = 116) with no/trace or mild MR and Group 2 (*n* = 60) with moderate or moderately severe MR. All patients had clinical and echocardiographic examinations, as well as a 24 h Holter ECG. *Results*: Group 2 had significantly more often the presence of the obstructive type of HCM (*p* < 0.001), syncope (*p* = 0.030), NYHA II class (*p* < 0.001), and atrial fibrillation (*p* = 0.023). Also, Group 2 had an enlarged left atrial dimension (*p* < 0.001), left atrial volume index (*p* < 0.001), and indirectly measured systolic pressure in the right ventricle (*p* < 0.001). Patients with a higher grade of MR had a significantly higher E/e′ (*p* < 0.001) and, as a result, higher values of Nt pro BNP values (*p* < 0.001) compared to Group 1. Kaplan–Meier analysis demonstrated that the event-free survival rate during a median follow-up of 88 (IQR 40–112) months was significantly higher in Group 1 compared to Group 2 (84% vs. 45% at 8 years; log-rank 20.4, *p* < 0.001). After adjustment for relevant confounders, the presence of moderate or moderately severe MR remained as an independent predictor of adverse outcomes (HR 2.788; 95% CI 1.221–6.364, *p* = 0.015). *Conclusions*: The presence of moderate or moderately severe MR was associated with unfavorable long-term outcomes in HCM patients.

## 1. Background

Hypertrophic cardiomyopathy (HCM) is defined as a condition characterized by increased thickness of the left ventricular (LV) walls or LV mass that is not caused by hypertension or heart valve diseases [1,2]. HCM has been a subject of interest and a challenge for cardiologists over the past fifty years. The prevalence of HCM is approximately 0.2%, or 1 in 500 of the general population, and it is one of the most common genetic cardiovascular diseases [1,3,4]. However, a large number of individuals who have a genetic mutation for HCM remain clinically undetected, making HCM rare in routine practice [5]. Nevertheless, HCM is the leading cause of sudden cardiac death in young individuals, including active athletes [6]. It is important to note that HCM is a significant cause of cardiovascular disability, including the development of heart failure, atrial fibrillation (AF), and ischemic stroke [6,7]. Interestingly and paradoxically, HCM can sometimes be of minimal or no clinical significance. In such cases, it is compatible with normal lifespan and longevity [4,6].

Studies examining the mitral valve in patients with HCM have shown that the mitral valve itself can be structurally altered [8,9]. Therefore, a detailed evaluation of the anatomy and function of the mitral valve, as well as the significance of mitral regurgitation (MR), is essential in patients with HCM [8,9,10,11]. The aim of our study is to examine the potential influence of moderate or moderately severe MR on the prognosis, clinical presentation, and structural characteristics of HCM patients.

## 2. Methods

From April 2008 until June 2021, we prospectively included 176 patients with primary HCM at the Clinic for Cardiology, University Clinical Center of Serbia. The patients fulfilled the following inclusion criteria: (1) an unexplained maximal wall thickness of ≥15 mm and a septum/posterior wall ratio > 1.5 in the absence of another cardiac or systemic cause of LV hypertrophy [1]; (2) preserved LV ejection fraction (>55%); and (3) clinical follow-up. Based on hemodynamic characteristics, asymmetric HCM was defined as non-obstructive and obstructive (HOCM) if there was a resting gradient ≥ 30 mmHg in the LV outflow tract [1].

The exclusion criteria for the study were: (1) a poor acoustic window for echo assessment; (2) New York Heart Association (NYHA) class III or IV; (3) the presence of any disease (e.g., neurological, cancer, or infectious) the severity of which is likely to contribute to a short life expectancy; (4) the presence of chronic renal insufficiency; (5) the presence of significant coronary artery stenosis (a quantitatively assessed coronary diameter reduction ≥ 50%) on coronary angiography or a history of coronary artery disease; and (6) those with more than mild aortic or mitral stenosis. All patients underwent a clinical examination and echocardiography, electrocardiography (ECG), and 24 h Holter ECG monitoring. Plasma levels of N-terminal pro B-type natriuretic peptide (Nt-pro-BNP) were obtained immediately before echocardiographic examination in all patients and were analyzed by the electrochemiluminescence immunoassay technique (ECLIA; Roche Diagnostics, Basel, Switzerland). Coronary angiography was performed in 119 patients who had either anginal symptoms or other indications outlined in the existing guidelines [2], and none of them had significant coronary stenosis. The remaining 57 patients had either less than a 5% probability of having coronary artery disease [12] or a negative stress echocardiography (SE) test [13].

### 2.1. Echocardiography

The echocardiographic examination was performed using the Acuson Sequoia C256 ultrasound system (Siemens Medical Solutions USA, Inc., Mountain View, CA, USA) and the GE Healthcare Vivid E9 ultrasound system (GE Vingmed Ultrasound AS, Horten, Norway) with multifrequency probes. M-mode and pulsed Doppler measurements were performed according to the current recommendations [14]. The following parameters were collected in M-mode in the parasternal long-axis view: LV end-diastolic dimension, LV end-systolic dimension, end-diastolic diameter of the left atrium (LA), and end-diastolic thickness of the septum and posterior wall. Additionally, LA volumes were measured using the modified Simpson biplane method [14]. LA volume was indexed to body surface area (LAVI), and increased LAVI was defined as greater than 34 mL/m^2^ [14]. The left ventricular outflow tract gradient (LVOTG) was assessed using color Doppler, pulsed and continuous-wave Doppler at rest, and during the Valsalva maneuver or SE in each patient. Early and late diastolic filling velocities of the LV (E and A) were measured at the tips of the mitral valve. Early (e′) and late (a′) diastolic velocities of the lateral mitral annulus were measured using pulsed Doppler from the standard four-chamber view. The ratio of early transmitral flow to the early diastolic velocity of the lateral mitral annulus (E/e′) was derived.

**Figure 1:**
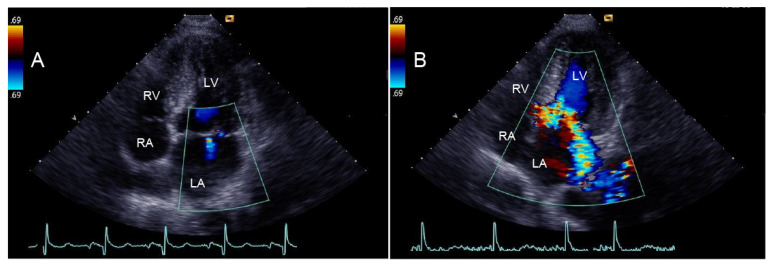
Examples of two patients with (**A**) mild and (**B**) moderately severe eccentric mitral regurgitation in four-chamber view. RA: right atrium, RV: right ventricle, LV: left ventricle, LA: left atrium.

The severity of MR was integratively assessed during routine echocardiographic evaluation according to the current guidelines [15]. Thus, the following criteria were employed as markers of mild MR: a small, narrow central jet; a vena contracta width < 3 mm; a dominant A-wave mitral inflow pattern; and a faint and incomplete signal jet by continuous wave Doppler [15]. Moderate and moderately severe/severe MR were defined on the basis of a wide regurgitant MR jet visualized by color flow Doppler, a complete (holosystolic) and dense continuous-wave Doppler MR jet trace, as well as a vena contracta width > 3 mm [15]. Another supportive sign of MR severity was an E-wave velocity greater then 1.2 m/s [15]. Accordingly, patients were classified as having MR graded as none (*n* = 17), mild (*n* = 99), moderate (*n* = 56), moderately severe (*n* = 4), or severe (*n* = 0) [15]. Significant MR was defined as greater than or equal to moderate MR [11,16] (Figure 1). Consequently, HCM patients were divided into two groups: Group 1 (*n* = 116), which included patients without MR or with trace/mild MR, and Group 2 (*n* = 60), which included patients with moderate or moderately severe MR.

### 2.2. Assessment of Outcomes

Follow-up was performed by an outpatient medical visit or telephone contact in all patients. In case of an adverse event, all hospital records were obtained. The primary outcome was a composite of: (1) HCM-related death, considered in the case of heart failure (occurring in the setting of cardiac decompensation, pulmonary edema, or a progressive course to end-stage disease), sudden cardiac death (including cardiac arrest with resuscitation after cardiac arrest), or fatal ischemic stroke; (2) heart failure requiring hospitalization (in the setting of pulmonary congestion on chest X-ray); (3) sustained ventricular tachycardia (VT) or appropriate shocks by an implanted defibrillator; and (4) ischemic stroke (judged to be a direct consequence of embolic events usually in the setting of paroxysmal or chronic AF). Any unexplained sudden death was regarded as cardiac and attributed to adverse events. All events were clinically adjudicated by the 2 senior cardiologists.

### 2.3. Statistical Analysis

All numeric data were expressed as means ± standard deviations (SDs), and all categorical data were expressed as frequencies or percentages. Differences in continuous variables were assessed with the Student’s *t*-test. Categorical data were compared using the chi-square test or Fisher’s exact test, as appropriate. To achieve a normal distribution of Nt pro BNP values and to compare between groups, natural logarithm values of Nt pro BNP were calculated. Survival rates were assessed using Kaplan–Meier curves and compared using the log-rank test. Univariate and multivariate Cox regression analysis was used to test the association of selected variables with patient outcomes. The univariate analysis included all available major clinical and echocardiographic parameters used to assess increased risk in HCM. Variables that were significantly associated with the primary outcome in the univariate analysis (*p* < 0.05) were included in the multivariate model. Hazard ratios (HRs) with corresponding 95% confidence intervals (CIs) were estimated. Statistical significance was defined as *p* < 0.05.

## 3. Results

We prospectively included 176 patients with primary HCM, of whom the majority were females (53%). Out of the total patient population, 129 individuals (73%) had asymmetrical non-obstructive HCM, while 47 patients (27%) had HOCM.

The demographic and clinical characteristics of the patients are presented in Table 1. The patients in Group 2 were significantly older (*p* < 0.001) in comparison to Group 1. Additionally, the female gender was more prevalent in Group 2 compared to Group 1 (*p* = 0.004). There were significantly more patients with arterial hypertension, syncope, and NYHA class II in Group 2, while there was no significant difference in family history of HCM and sudden cardiac death (SCD) among the study groups. Patients with higher degrees of MR had a higher frequency of AF (*p* = 0.023) compared to patients with mild MR, while there was no significant difference in the presence of unsustained ventricular tachycardia on 24 h Holter ECG. Concerning medical treatment, there were no differences between the groups, except for the use of diuretics, which was more frequent in patients with more severe MR.

The echocardiographic parameters are presented in Table 2. The study groups did not demonstrate any statistically significant differences in the end-diastolic and end-systolic dimensions of LV, ejection fraction, right ventricular dimension, interventricular septum thickness, or maximum LV wall dimension. Patients with higher degrees of MR had significantly more systolic anterior motion (SAM), resulting in higher resting and provoked LVOTG and more frequent eccentric MR jets. Also, the presence of calcified mitral annulus, enlarged antero-posterior dimension of the left atrium (LA), left atrial volume index (LAVI) (*p* < 0.001), and indirectly measured right ventricular systolic pressure (RVSP) (*p* < 0.001) were higher in Group 2. Consequently, patients with more severe MR had significantly higher values of LV inflow, including higher E- and A-wave values and E/e′ ratios (*p* < 0.001), and consequently higher values of N-terminal pro B-type natriuretic peptide (NT-proBNP) (*p* < 0.001) in comparison to Group 1.

During a median follow-up of 88 months (interquartile range (IQR) (40–112)), the primary composite outcome occurred in 44/176 (25%) patients. In Group 1, 16/116 patients (13.7%) experienced adverse events, including cardiac death in 8 patients (SCD in 3, 2 were due to heart failure, and 3 were a result of ischemic stroke), hospitalization for heart failure in 4 patients, ischemic stroke in 2 patients, and sustained VT in 2 patients. However, in Group 2, there were 28/60 patients (46.7%, *p* < 0.001, compared to Group 1) who experienced adverse events, with cardiac death identified in 13 patients (SCD in 6, 3 were due to heart failure, and 4 were a result of ischemic stroke), hospitalization for heart failure in 12 patients, ischemic stroke in 1 patient, and sustained VT in 2 patients. Thus, HCM-related cardiac death was more prevalent in Group 2 in comparison to Group 1 (13 (22%) vs. 8 (7%), *p* = 0.005, respectively). Additionally, the prevalence of heart failure (both fatal and non-fatal) was significantly higher in Group 2 compared to Group 1 (15 (25%) vs. 6 (5.2%), *p* < 0.001, respectively). However, there was no significant difference in the occurrence of ischemic stroke or sustained VT between the two groups. Also, during follow-up, new onset of AF was significantly higher in Group 2 in comparison to Group 1 (12 (20%) vs. 4 (3.5%), *p* < 0.001, respectively).

**Figure 2:**
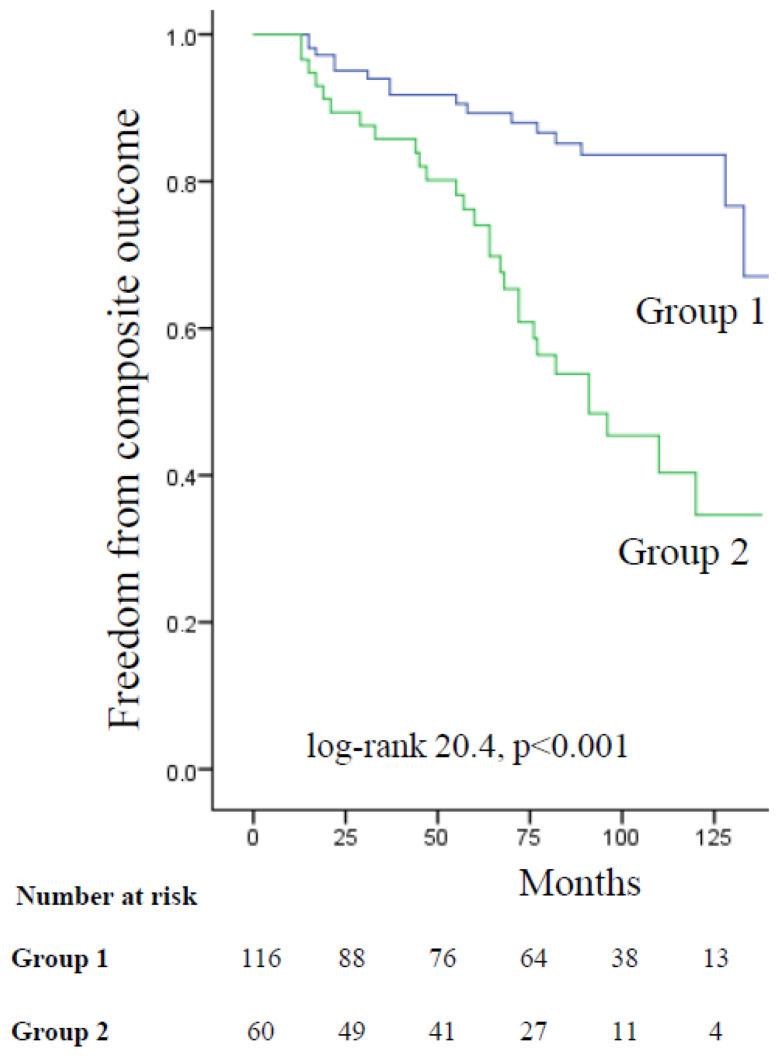
Kaplan–Meier survival curves for the composite outcome according to the severity of mitral regurgitation (MR). Group 1: patients without MR or with trace/mild MR, Group 2: patients with moderate or moderately severe MR.

By Kaplan–Meier analysis for the primary composite outcome (Figure 2), the patients in Group 1 had a significantly higher cumulative survival rate without adverse events compared to the patients in Group 2 (84% vs. 45% at 8 years; log-rank 20.4, *p* < 0.001).

Univariable Cox proportional hazard regression analysis showed that female sex, age, AF on Holter ECG, maximal induced LVOTG ≥ 50 mm Hg, presence of LAVI > 34 mL/m^2^, and moderate/moderately severe MR were all significantly associated with the primary outcome (Table 3). However, multivariable analysis identified only the presence of moderate or moderately severe MR as an independent predictor for adverse cardiac outcomes (HR 2.788; 95% CI 1.221–6.364, *p* = 0.015) (Table 4). Furthermore, in multivariate analysis, the presence of moderate or moderately severe MR remained an independent predictor for adverse cardiac outcomes even in the subgroup of patients with the non-obstructive form of HCM (HR 3.046; 95% CI 1.282–7.236, *p* = 0.012).

## 4. Discussion

Our study has shown that the presence of moderate or moderately severe MR at rest is an independent and strong predictor of unfavorable long-term outcomes in patients with HCM. Additionally, the presence of moderate or moderately severe MR is indicative of an increased risk for the development of heart failure but also of HCM-related cardiac death. Therefore, MR can be considered as an additional marker of an unfavorable prognosis, along with well-known clinical factors (age, gender, family history of sudden cardiac death, presence of syncope, and NSVT) and echocardiographic markers (presence of a maximum LVOTG ≥ 50 mmHg, increased LAVI, maximum LV wall thickness, presence of massive hypertrophy (≥30 mm), and impaired coronary flow velocity reserve) [1,7,17,18,19,20,21,22,23]. Patients with a higher degree of MR demonstrated more severe clinical symptoms and structural changes compared to those with mild MR.

As presented in our study, in patients with HOCM, LVOTG is usually induced by mitral valve SAM and septal contact due to flow drag, resulting in more severe MR in comparison to the non-obstructive HCM [6,24]. LVOTG in HCM is typically labile, and its magnitude can change spontaneously, after alcohol intake or a large meal and during physical activity. Thus, obstruction can be induced by hemodynamic changes provoked by the inhalation of amyl nitrate, the Valsalva maneuver, infusion of positive inotropic drugs, or during exercise stress testing [11,22,25]. Significant LVOTG at rest (gradients ≥ 30 mm Hg) is present in approximately 25% of affected individuals [20]. Furthermore, a recent study by Maron et al. has shown that the prevalence of inducible LV outflow tract obstruction in cohorts of patients evaluated at referral centers can be as high as 70% [6,26].

Two-dimensional echocardiography can reveal structural changes in the mitral valve, including prolapse, excessive leaflet tissue, elongated chords, marked mitral annular calcification, elongated mitral leaflets (with coaptation at the leaflet body rather than at the tip), anterior displacement of the mitral apparatus, and direct attachment of the papillary muscle to the anterior leaflet of the mitral valve [27]. Anterior displacement of the papillary muscle is the most important primary structural change in the mitral apparatus that leads to the development of obstruction in the LV outflow tract [28].

Two conditions are responsible for the development of SAM: (1) a pathological valvular apparatus with leaflets of sufficient size to induce their motion and (2) the presence of drag forces which draw both the mitral leaflets and chordae towards the interventricular septum causing leaflet–septal contact and obstruction at this site [24]. As a result of SAM of the anterior mitral leaflet and failure of the posterior leaflet to move forward as much as the anterior leaflet, incomplete leaflet coaptation results in posteriorly directed MR [24,27]. The presence of central or anteriorly directed MR raises the suspicion of structural disease of the mitral valve [24,27]. Furthermore, repeated contact between the mitral leaflet and the septum causes mechanical trauma, leading to the thickening and fibrosis of the leaflets and chordae tendineae, which can pose a significant risk for chordal rupture or infective endocarditis [8,10].

NYHA functional class II was present more often in Group 2 in comparison to Group 1. Although we included in the study patients who were less symptomatic (there were no patients with NYHA functional classes III and IV), our findings may indicate the contribution of moderate or moderately severe MR in the development of heart failure during follow-up. Supposedly, the presence of at least moderate MR can be a risk factor for heart failure aggravation through the elevation of LV filling pressure in the hypertrophied and stiff myocardium [16,29]. Additionally, the presence of moderate or moderately severe MR may also be directly related to the ventricular remodeling with chronic volume overload and progressive deterioration of myocardial function, leading to the development of heart failure and cardiac death [16]. Additionally, there was a statistically significant difference in the presence of a calcified mitral annulus in Group 2, which may also contribute to increased MR, as previously shown, since marked calcified mitral annulus itself induces anterior displacement of the mitral leaflet, causing LV outflow tract obstruction [16,27]. Furthermore, we demonstrated that even in the subgroup of non-obstructive HCM patients there was a significant association between the presence of moderate or moderately severe MR and clinical prognosis, emphasizing the importance of MR evaluation in this population. Our findings are in line with a recent study of East Asian patients predominantly with the non-obstructive type of HCM, which showed that the presence of greater than or equal to moderate MR is associated with an unfavorable prognosis [16]. Additionally, the authors showed that progression of MR was an independent prognostic factor of clinical outcomes, along with female sex, AF, and larger LAVI [16]. In another primary exercise echocardiography study that included asymptomatic HCM patients, the authors showed that resting and even post-stress MR were not predictive of long-term outcomes [30]. One potential reason for this result is that these patients represented an asymptomatic HCM cohort who were able to undergo exercise echocardiography, since the aim was to present the value of reduced exercise capacity in the prognosis of HCM patients.

The pathological substrate for arrhythmias in HCM is the disorganized cellular architecture and fibrosis of myocardial cells [31]. Triggers for the development of ventricular arrhythmias include ischemia, LVOTG, physical exercise, and excessive sympathetic stimulation [6,25]. In our HCM group, no significant difference was observed in the presence of NSVT between the two groups of subjects. However, AF was statistically more common in patients with a higher degree of MR as well as new onset of AF during follow-up. AF is furthermore associated with LA dilation, which is associated with more significant MR and LVOTG, as demonstrated in our group of patients [32]. Ischemic stroke is the most important sequela of AF, warranting a low threshold for prophylaxis with vitamin K antagonists or novel direct oral agents [1]. Contrary to some previous reports [32], in our study, AF was not a predictor of adverse outcomes.

The levels of NT-pro BNP were significantly higher in the group with more severe MR, which may indicate the contribution of MR together with LVOTG in the development of elevated left ventricular filling pressure in these patients. Furthermore, the reduction in chamber compliance and increased chamber stiffness occur due to increased LV mass, myocardial fibrosis, and ischemia [7,33]. The E/e′ ratio of the lateral mitral annulus has been shown to be a reasonably accurate non-invasive predictor of elevated LV filling pressure [34,35,36], since conventional Doppler parameters, such as the E-wave deceleration time and the E/A ratio of transmitral flow, do not correlate well with LV end-diastolic pressure in HCM [37]. The values of E/e′ of the lateral mitral annulus were significantly higher in the group with more significant MR, indicating the contribution of MR to the existing substrate of diastolic dysfunction for associated symptoms in this patient population.

Disease progression in HCM is often due to microvascular and diastolic dysfunction and the presence of significant LVOTG and MR [4]. All the above processes result in a reduction in exercise capacity and could ultimately progress to congestive heart failure and death [1,7,29,33,36]. Thus, the relief of LV outflow tract obstruction may cause a reduction in MR severity and less vasodilatory reserve to be exhausted at rest, in addition to reductions in wall stress and extravascular compression [24,38]. Pharmacological treatment with beta blockers represents the first line of the management of symptomatic HOCM patients [1,2]. Furthermore, novel medical therapies in HCM are evolving with emerging pharmacological options for HOCM, including mavacamten—an allosteric modulator of cardiac myosin and strong negative inotrope that reduces LV contractility and consequently LVOTG and MR severity and possibly HF symptoms [39]. What is of the utmost importance is detailed assessment of the mitral valve, especially in those who are highly symptomatic and with significant LVOTG. In symptomatic HCM patients, invasive therapies to relieve LV outflow tract obstruction (surgical myectomy with or without mitral valve surgery or alcohol septal ablation) are associated with excellent long-term outcomes [1,2,27]. In association with myectomy, the replacement, remodeling, or repair of the mitral valve apparatus and submitral structures to relieve LV outflow tract obstruction and MR may be performed [1,2,27].

## 5. Study Limitations

In the study, we enrolled patients with no or mild symptoms, but during follow-up one alcohol septal ablation, one surgical myectomy, and two mitral valve replacements with myectomy occurred. All procedures, except the surgical myectomy, were performed in the group with more severe MR. Thus, although infrequent, these procedures might have influenced the outcomes and, furthermore, the potential prognostic value of MR in the group with more severe MR.

Effective regurgitant orifice area assessment and MR volume quantification using the proximal isovelocity surface area method were not performed. This method is less accurate in SAM-related MR eccentric jets typical for HOCM [15].

## 6. Conclusions

The presence of moderate or moderately severe MR has been associated with poor long-term outcomes in HCM patients. A higher degree of MR is related to disease severity in terms of structural, clinical, or arrhythmogenic aspects. Our study emphasizes the importance of the comprehensive evaluation of MR severity and structural changes of the mitral valve. Therefore, the identification of patients with higher degrees of MR might be of great clinical value in order to improve the risk stratification of HCM patients.

## Figures and Tables

**Table 1:** Clinical characteristics of patients.

Variables	Total (*n* = 176)	Group 1 (*n* = 116)	Group 2 (*n* = 60)	*p* Value Group 1 vs. Group 2
Age—years	48 ± 15	45 ± 14	54 ± 15	<0.001
BSA—m^2^	1.85 ± 0.2	1.87 ± 0.2	1.82 ± 0.17	0.06
Female sex—no. (%)	94 (53)	53 (46)	41 (68)	0.004
Hypertension—no. (%)	61 (35)	33 (28)	28 (47)	0.016
Syncope—no. (%)	22 (12)	10 (9)	12 (20)	0.03
Family history of HCM—no. (%)	67 (38)	48 (41)	19 (32)	0.208
Family history of SCD—no. (%)	25 (14)	17 (15)	8 (13.3)	0.812
NYHA functional class—no. (%)				<0.001
I	110 (62)	91 (78)	19 (32)	
II	66 (37)	25 (22)	41 (68)	
Unsustained ventricular tachycardia on Holter ECG—no. (%)	36 (21)	28 (25)	8 (14)	0.091
Atrial fibrillation on Holter ECG—no. (%)	31 (18)	15 (13)	16 (27)	0.023
Diastolic blood pressure—mmHg	77 ± 8	78 ± 8	78 ± 9	0.811
Systolic blood pressure—mmHg	120 ± 15	119 ± 15	120 ± 15	0.658
Baseline heart rate—beats/min	69 ± 14	70 ± 14	68 ± 14	0.482
Medical therapy—no. (%)				
Beta blockers	150 (85)	95 (82)	55 (92)	0.083
Ca antagonists	32 (18)	17 (15)	15 (25)	0.092
ACEI/ARB	47 (27)	27 (23)	20 (33)	0.153
Diuretic	33 (19)	15 (13)	18 (30)	0.006
Amiodarone	24 (14)	12 (10)	12 (20)	0.077

Plus–minus values are means ± SDs. BSA: body surface area, HCM: hypertrophic cardiomyopathy, SCD: sudden cardiac death, ECG: electrocardiogram, NYHA: New York Heart Association, ACEI: angiotensin-converting enzyme inhibitors, ARB: angiotensin II receptor blockers.

**Table 2:** Echocardiographic parameters.

Variables	Total (*n* = 176)	Group 1 (*n* = 116)	Group 2 (*n* = 60)	*p* Value Group 1 vs. Group 2
LV end-diastolic dimension—mm	46 ± 5	45 ± 5	46 ± 5	0.099
LV end-systolic dimension—mm	27 ± 5	28 ± 5	27 ± 4	0.089
IVS thickness—mm	19 ± 4	19 ± 4	20 ± 4	0.339
PW thickness—mm	10 ± 2	9.8 ± 2	11 ± 3	<0.001
IVS/PW ratio	1.96 ± 0.57	2.0 ± 0.53	1.8 ± 0.62	0.039
Maximal wall thickness—mm	21 ± 5	21 ± 5	22 ± 4	0.099
LV wall thickness ≥ 30 mm—no. (%)	8 (5)	4 (3)	4 (7)	0.331
LV ejection fraction—%	70 ± 8	69 ± 8	70 ± 8	0.222
LVOTG at rest—median (IQR)—mmHg	10 (6–30)	7 (6–12)	36 (12–63)	<0.001
LVOTG at rest ≥ 30 mmHg—no. (%)	47 (27)	13 (11)	34 (57)	<0.001
Maximal induced LVOTG ≥ 50 mmHg—no. (%)	44 (25)	9 (8)	35 (58)	<0.001
Left atrial dimension—mm	43 ± 6	41 ± 6	45 ± 6	<0.001
LAVI—mL/m^2^	38 ± 14	34 ± 12	45 ± 16	<0.001
LAVI > 34 mL/m^2^—br. (%)	94 (53)	46 (40)	48 (80)	<0.001
RVSP—mmHg	34 ± 9	32 ± 7	38 ± 10	<0.001
E-wave—m/s	0.73 ± 0.20	0.68 ± 0.17	0.81 ± 0.25	<0.001
A-wave—m/s	0.67 ± 0.26	0.59 ± 0.18	0.82 ± 0.31	<0.001
E/A	1.26 ± 0.69	1.31 ± 0.69	1.16 ± 0.69	0.192
Mitral lateral annular e′—m/s	0.103 ± 0.033	0.110 ± 0.033	0.088 ± 0.026	<0.001
Mitral lateral annular a′—m/s	0.112 ± 0.036	0.110 ± 0.034	0.088 ± 0.026	0.286
E/e′	7.640 ± 3.036	6.58 ± 2.27	9.68 ± 3.29	<0.001
Ln NT-pro-BNP—pg/mL	6.88 ± 0.99	6.63 ± 0.95	7.37 ± 0.86	<0.001
Eccentric jet of MR—no. (%)	52 (30)	12 (10)	40 (67)	<0.001
Systolic anterior motion—no. (%)	74 (42)	29 (25)	45 (75)	<0.001
Mitral annular calcification—no. (%)	29 (17)	9 (8)	20 (33)	<0.001

Plus–minus values are means ± SDs. LV: left ventricular, IVS: interventricular septum, PW: posterior wall, LAVI: left atrial volume indexed for body surface area, LVOTG: left ventricular outflow tract gradient, IQR: interquartile range, RVSP: right ventricular systolic pressure.

**Table 3:** Univariable prognostic predictors of the composite outcome.

Variables	Univariable Analysis		
	HR	*p* Value	95% CI
Female sex	2.494	0.007	1.284–4.845
Age—years	1.027	0.018	1.005–1.050
Family history of SCD	1.833	0.122	0.850–3.952
Atrial fibrillation on Holter ECG	2.269	0.011	1.211–4.252
NSVT on Holter ECG	1.409	0.329	0.708–2.804
Syncope	0.756	0.556	0.298–1.920
Maximal wall thickness—mm	1.056	0.066	0.996–1.119
LV wall thickness ≥ 30 mm	1.365	0.603	0.422–4.420
Maximal induced LVOTG ≥ 50 mmHg	1.949	0.031	1.061–3.580
Moderate or moderately severe MR	3.758	<0.001	2.028–6.964
LAVI > 34 mL/m^2^	2.578	0.005	1.341–4.954

HR: hazard ratio, CI: confidence interval, SCD: sudden cardiac death, AF: atrial fibrillation, NSVT: non-sustained ventricular tachycardia, LV: left ventricular, LAVI: left atrial volume indexed for body surface area, LVOTG: left ventricular outflow tract gradient, MR: mitral regurgitation.

**Table 4:** Multivariable prognostic predictors of the composite outcome.

Variables	Multivariable Analysis		
	HR	*p* Value	95% CI
Female sex	1.940	0.057	0.981–3.836
Age—years	1.000	0.987	0.976–1.025
Atrial fibrillation on Holter ECG	1.640	0.157	0.827–3.253
LAVI > 34 mL/m^2^	1.546	0.248	0.738–3.239
Maximal induced LVOTG ≥ 50 mmHg	0.889	0.759	0.421–1.878
Moderate or moderately severe MR	2.788	0.015	1.221–6.364

HR: hazard ratio, CI: confidence interval, AF: atrial fibrillation, LV: left ventricular, LAVI: left atrial volume indexed for body surface area, LVOTG: left ventricular outflow tract gradient, MR: mitral regurgitation.

## Data Availability

The data presented in this study are available on request from the corresponding author.

## References

[1] Ommen S.R., Mital S., Burke M.A., Day S.M., Deswal A., Elliott P., Evanovich L.L., Hung J., Joglar J.A., Kantor P. (2020). 2020 AHA/ACC Guideline for the Diagnosis and Treatment of Patients with Hypertrophic Cardiomyopathy: Executive Summary: A Report of the American College of Cardiology/American Heart Association Joint Committee on Clinical Practice Guidelines. J. Am. Coll. Cardiol..

[2] Elliott P.M., Anastasakis A., Borger M.A., Borggrefe M., Cecchi F., Charron P., Hagege A.A., Lafont A., Limongelli G., Mahrholdt H. (2014). 2014 ESC Guidelines on diagnosis and management of hypertrophic cardiomyopathy: The Task Force for the Diagnosis and Management of Hypertrophic Cardiomyopathy of the European Society of Cardiology (ESC). Eur. Heart J..

[3] Velicki L., Jakovljevic D.G., Preveden A., Golubovic M., Bjelobrk M., Ilic A., Stojsic S., Barlocco F., Tafelmeier M., Okwose N. (2020). Genetic determinants of clinical phenotype in hypertrophic cardiomyopathy. BMC Cardiovasc. Disord..

[4] Popa-Fotea N.M., Micheu M.M., Bataila V., Scafa-Udriste A., Dorobantu L., Scarlatescu A.I., Zamfir D., Stoian M., Onciul S., Dorobantu M. (2019). Exploring the Continuum of Hypertrophic Cardiomyopathy—From DNA to Clinical Expression. Medicina.

[5] Maron B.J., Peterson E.E., Maron M.S., Peterson J.E. (1994). Prevalence of hypertrophic cardiomyopathy in an outpatient population referred for echocardiographic study. Am. J. Cardiol..

[6] Maron B.J. (2018). Clinical Course and Management of Hypertrophic Cardiomyopathy. N. Engl. J. Med..

[7] Tesic M., Djordjevic-Dikic A., Giga V., Stepanovic J., Dobric M., Jovanovic I., Petrovic M., Mehmedbegovic Z., Milasinovic D., Dedovic V. (2021). Prognostic Value of Transthoracic Doppler Echocardiography Coronary Flow Velocity Reserve in Patients with Asymmetric Hypertrophic Cardiomyopathy. J. Am. Heart Assoc..

[8] Klues H.G., Maron B.J., Dollar A.L., Roberts W.C. (1992). Diversity of structural mitral valve alterations in hypertrophic cardiomyopathy. Circulation.

[9] Molisana M., Selimi A., Gizzi G., D’Agostino S., Ianni U., Parato V.M. (2022). Different mechanisms of mitral regurgitation in hypertrophic cardiomyopathy: A clinical case and literature review. Front. Cardiovasc. Med..

[10] Klues H.G., Roberts W.C., Maron B.J. (1991). Anomalous insertion of papillary muscle directly into anterior mitral leaflet in hypertrophic cardiomyopathy. Significance in producing left ventricular outflow obstruction. Circulation.

[11] Peteiro J., Bouzas-Mosquera A., Fernandez X., Monserrat L., Pazos P., Estevez-Loureiro R., Castro-Beiras A. (2012). Prognostic value of exercise echocardiography in patients with hypertrophic cardiomyopathy. J. Am. Soc. Echocardiogr..

[12] Diamond G.A., Forrester J.S. (1979). Analysis of probability as an aid in the clinical diagnosis of coronary-artery disease. N. Engl. J. Med..

[13] Lazzeroni E., Picano E., Dodi C., Morozzi L., Chiriatti G.P., Lu C., Botti G., Eeho-Persantine International Cooperative (EPIC) Study Group—Subproject Hypertrophic Cardiomyopathy (1995). Dipyridamole echocardiography for diagnosis of coexistent coronary artery disease in hypertrophic cardiomyopathy. Echo-Persantine International Cooperative (EPIC) Study Group--Subproject Hypertrophic Cardiomyopathy. Am. J. Cardiol..

[14] Lang R.M., Badano L.P., Mor-Avi V., Afilalo J., Armstrong A., Ernande L., Flachskampf F.A., Foster E., Goldstein S.A., Kuznetsova T. (2015). Recommendations for cardiac chamber quantification by echocardiography in adults: An update from the American Society of Echocardiography and the European Association of Cardiovascular Imaging. J. Am. Soc. Echocardiogr..

[15] Zoghbi W.A., Adams D., Bonow R.O., Enriquez-Sarano M., Foster E., Grayburn P.A., Hahn R.T., Han Y., Hung J., Lang R.M. (2017). Recommendations for Noninvasive Evaluation of Native Valvular Regurgitation: A Report from the American Society of Echocardiography Developed in Collaboration with the Society for Cardiovascular Magnetic Resonance. J. Am. Soc. Echocardiogr..

[16] Kim D.Y., Seo J., Cho I., Hong G.R., Ha J.W., Shim C.Y. (2023). Prognostic Implication of Mitral Valve Disease and Its Progression in East Asian Patients with Hypertrophic Cardiomyopathy. J. Am. Heart Assoc..

[17] Spirito P., Autore C., Rapezzi C., Bernabò P., Badagliacca R., Maron M.S., Bongioanni S., Coccolo F., Estes N.M., Barillà C.S. (2009). Syncope and risk of sudden death in hypertrophic cardiomyopathy. Circulation.

[18] Losi M.-A., Betocchi S., Barbati G., Parisi V., Tocchetti C.-G., Pastore F., Migliore T., Contaldi C., Caputi A., Romano R. (2009). Prognostic significance of left atrial volume dilatation in patients with hypertrophic cardiomyopathy. J. Am. Soc. Echocardiogr..

[19] Adabag A.S., Casey S.A., Kuskowski M.A., Zenovich A.G., Maron B.J. (2005). Spectrum and prognostic significance of arrhythmias on ambulatory Holter electrocardiogram in hypertrophic cardiomyopathy. J. Am. Coll. Cardiol..

[20] Maron M.S., Olivotto I., Betocchi S., Casey S.A., Lesser J.R., Losi M.A., Cecchi F., Maron B.J. (2003). Effect of left ventricular outflow tract obstruction on clinical outcome in hypertrophic cardiomyopathy. N. Engl. J. Med..

[21] Elliott P.M., Gimeno Blanes J.R., Mahon N.G., Poloniecki J.D., McKenna W.J. (2001). Relation between severity of left-ventricular hypertrophy and prognosis in patients with hypertrophic cardiomyopathy. Lancet.

[22] Ciampi Q., Olivotto I., Gardini C., Mori F., Peteiro J., Monserrat L., Fernandez X., Cortigiani L., Rigo F., Lopes L.R. (2016). Prognostic role of stress echocardiography in hypertrophic cardiomyopathy: The International Stress Echo Registry. Int. J. Cardiol..

[23] Preveden A., Golubovic M., Bjelobrk M., Miljkovic T., Ilic A., Stojsic S., Gajic D., Glavaski M., Maier L.S., Okwose N. (2022). Gender Related Differences in the Clinical Presentation of Hypertrophic Cardiomyopathy—An Analysis from the SILICOFCM Database. Medicina.

[24] Yu E.H., Omran A.S., Wigle E.D., Williams W.G., Siu S.C., Rakowski H. (2000). Mitral regurgitation in hypertrophic obstructive cardiomyopathy: Relationship to obstruction and relief with myectomy. J. Am. Coll. Cardiol..

[25] Ciampi Q., Olivotto I., Peteiro J., D’alfonso M.G., Mori F., Tassetti L., Milazzo A., Monserrat L., Fernandez X., Pálinkás A. (2021). Prognostic Value of Reduced Heart Rate Reserve during Exercise in Hypertrophic Cardiomyopathy. J. Clin. Med..

[26] Maron M.S., Link M.S., Udelson J.E., Kuvin J.T., Pandian N.G., Olivotto I., Nistri S., Cecchi F., Maron B.J., Zenovich A.G. (2006). Hypertrophic cardiomyopathy is predominantly a disease of left ventricular outflow tract obstruction. Circulation.

[27] Sherrid M.V., Balaram S., Kim B., Axel L., Swistel D.G. (2016). The Mitral Valve in Obstructive Hypertrophic Cardiomyopathy: A Test in Context. J. Am. Coll. Cardiol..

[28] Levine R.A., Vlahakes G.J., Lefebvre X., Guerrero J.L., Cape E.G., Yoganathan A.P., Weyman A.E. (1995). Papillary muscle displacement causes systolic anterior motion of the mitral valve. Experimental validation and insights into the mechanism of subaortic obstruction. Circulation.

[29] Palinkas E.D., Re F., Peteiro J., Tesic M., Palinkas A., Torres M.A.R., Dikic A.D., Beleslin B., Van De Heyning C.M., D’Alfonso M.G. (2022). Pulmonary congestion during Exercise stress Echocardiography in Hypertrophic Cardiomyopathy. Int. J. Cardiovasc. Imaging.

[30] Desai M.Y., Bhonsale A., Patel P., Naji P., Smedira N.G., Thamilarasan M., Lytle B.W., Lever H.M. (2014). Exercise echocardiography in asymptomatic HCM: Exercise capacity, and not LV outflow tract gradient predicts long-term outcomes. JACC Cardiovasc. Imaging.

[31] Hughes S.E. (2004). The pathology of hypertrophic cardiomyopathy. Histopathology.

[32] Olivotto I., Cecchi F., Casey S.A., Dolara A., Traverse J.H., Maron B.J. (2001). Impact of atrial fibrillation on the clinical course of hypertrophic cardiomyopathy. Circulation.

[33] Maron B.J., Rowin E.J., Udelson J.E., Maron M.S. (2018). Clinical Spectrum and Management of Heart Failure in Hypertrophic Cardiomyopathy. JACC Heart Fail..

[34] Nagueh S.F., Appleton C.P., Gillebert T.C., Marino P.N., Oh J.K., Smiseth O.A., Waggoner A.D., Flachskampf F.A., Pellikka P.A., Evangelisa A. (2009). Recommendations for the evaluation of left ventricular diastolic function by echocardiography. Eur. J. Echocardiogr..

[35] Nagueh S.F., Lakkis N.M., Middleton K.J., Spencer W.H., Zoghbi W.A., Quinones M.A. (1999). Doppler estimation of left ventricular filling pressures in patients with hypertrophic cardiomyopathy. Circulation.

[36] Tesic M., Seferovic J., Trifunovic D., Djordjevic-Dikic A., Giga V., Jovanovic I., Petrovic O., Marinkovic J., Stankovic S., Stepanovic J. (2017). N-terminal pro-brain natriuretic peptide is related with coronary flow velocity reserve and diastolic dysfunction in patients with asymmetric hypertrophic cardiomyopathy. J. Cardiol..

[37] Williams L.K., Frenneaux M.P., Steeds R.P. (2009). Echocardiography in hypertrophic cardiomyopathy diagnosis, prognosis, and role in management. Eur. J. Echocardiogr..

[38] Tesic M., Djordjevic-Dikic A., Beleslin B., Trifunovic D., Giga V., Marinkovic J., Petrovic O., Petrovic M., Stepanovic J., Dobric M. (2013). Regional difference of microcirculation in patients with asymmetric hypertrophic cardiomyopathy: Transthoracic Doppler coronary flow velocity reserve analysis. J. Am. Soc. Echocardiogr..

[39] Olivotto I., Oreziak A., Barriales-Villa R., Abraham T.P., Masri A., Garcia-Pavia P., Saberi S., Lakdawala N.K., Wheeler M.T., Owens A. (2020). Mavacamten for treatment of symptomatic obstructive hypertrophic cardiomyopathy (EXPLORER-HCM): A randomised, double-blind, placebo-controlled, phase 3 trial. Lancet.

